# Low-cost robotics and AI technologies for rehabilitation and health monitoring

**DOI:** 10.3389/frobt.2026.1919797

**Published:** 2026-10-05

**Authors:** João Silva Sequeira

**Affiliations:** Institute for Systems and Robotics, Instituto Superior Técnico, Lisbon, Portugal

**Keywords:** diabetic foot, epilepsy traits, machine learning, rehabilitation taxonomies, robot cane, robot kinematics, thermographic images

## Abstract

This paper reviews robotics technologies applied to rehabilitation. Besides a literature review, selected to highlight relevant topics/aspects, the paper presents a few examples of low cost technologies (which are particularly important in ageing demographics and struggling economies) and discusses applications in the overlap between rehabilitation and regular health monitoring. The purpose of this study is to frame rehabilitation robotics in terms of the existing technologies and to showcase low cost demonstrators with relevance in rehabilitation scenarios. Besides the encouraging results, obtained in real-world tests (a distinctive characteristic of the paper), the role of AI techniques in the development of low cost devices is also highlighted. Moreover, the paper also discusses the influence of human factors in the acceptance and adoption of alternative/disruptive technologies.

## 1 Introduction

Motion deficits in humans have often severe impacts in the quality of life. The constant scientific and technological developments steadily push rehabilitation technologies and respective practices. It is naturally an interdisciplinary area, where multiple technologies intersect each others and often show multiple alternative solutions.

Moreover, this match has close connections to human augmentation, an area where the aim is to extend the capabilities of the natural body using artificial structures (that might not need to comply with kinematic limitations of the human body). The recent advances in humanoid robotics showcase kinematics that can be adapted to people with severe locomotion deficits, e.g., paraplegic, to restore almost normal mobility. Rehabilitation aims at minimizing the effects of such deficits Sciascia and Cromwell (2012) by carefully analysing the kinematic chains involved in the movement.

The use of kinematic structures, namely, structures matching biomechanics, in rehabilitation has long been used, both for humans and animals. The study of the movement capabilities falls into the Robotics area and has been extensively studied in multiple texts (see, for instance, Cardona and Serrano (2025)).

The examples above straightforwardly suggest a bias of the field towards the minimization of physical deficits, i.e., in locomotion or manipulation. However, rehabilitation of mental/neurological deficits is becoming influential in quality of life. The process of neurological rehabilitation, as shown in Playford and Nair (2018), has clear intersections with physical rehabilitation, namely, in the physical connections, i.e., the devices tailored to reduce the deficits effects.

The broad purpose of this study is to raise awareness for the prospective impact of low cost technologies in rehabilitation. The paper frames rehabilitation robotics in terms of the existing technologies in Section 2 where taxonomies for rehabilitation devices and literature are reviewed. Low cost demonstrators with relevance in rehabilitation scenarios are discussed in Sections 3–5 with a summary of case studies. Section 6 discusses real world experimenting, human factors, and points to the big challenge which is the slow adoption of innovation in the healthcare domain.

## 2 Taxonomies for rehabilitation devices and literature review

Taxonomies for rehabilitation treatments have been proposed, trying to accommodate all aspects involved in specific areas of intervention, Dijkers et al. (2014). Their usefulness lies in the structured/rationalised/standardised approach they provide, not only for treatment and therapeutics but also to system design (on a more general view of taxonomies see, for instance, Bozeman and McKelvey (1983)).

For physical rehabilitation devices, simple approaches only consider exoskeletons and end-effector devices, Lee et al. (2020). However, in what concerns walking aids, one can identify (i) robotic wheelchairs, Sivakanthan et al. (2022), (ii) robotic walkers and smart canes, Martins et al. (2012), and (iii) robotic wearables, Lebleu et al. (2024); Cha et al. (2025). Each of these can be further classified in terms of level of automation, the type of human interaction, and even the respective TRL.

Within functional classes, multiple taxonomies have also been proposed in the literature, e.g., “walking support systems for the elderly” in Noriyuki, (2000), “walking assistance systems”, in Van der Loos and Reinkensmeyer (2008), or “mobility assistance devices” in Speich and Rosen (2004).

For mental/neural rehabilitation, Lerín-Calvo et al. (2026) recognize the need for structured frameworks (taxonomies) that link theoretical models and practice. Aspects related to stability, muscle performance, and neuromuscular control/coordination, among other, are directly connected to rehabilitation devices and to the technology areas, namely, sensors, mechanisms are the respective actuators, and device control.

Rehabilitation is an area in the intersection of multiple areas of technology, ranging from engineering to social sciences. Essentially, it is the re-training of physical skills to reduce/minimize deficits in motion and/or mental processes. This re-training is a feedback process, strongly dependent of observation and actuation, and hence technology related to sensors, is naturally relevant. Moreover, virtually all technologies can be used in rehabilitation. The overlapping between rehabilitation and observation/inspection/monitoring arises through the sensors domain. The Action-Observation Treatment paradigm, see for instance Buccino (2014), is a clear example of the connection between the two areas, not only in rehabilitation but in therapeutics. Technologies developed primarily for observation are often also used to monitor the progress of rehabilitation and adjustment of therapeutics. In addition, while the cost of rehabilitation processes tends to be (very) high due to the nature of the devices involved, it is important that monitoring costs be kept as small as possible. Furthermore, some authors argue that multiple barriers to the adoption of rehabilitation robotics technologies still exist, Beani et al. (2026), e.g., cost, adaptation/alignment to the real needs of patients, and the lack of engagement by some stakeholders. Co-creation with end-users is deemed to be pivotal.

The literature on rehabilitation and, specifically, the associated technologies is growing consistently, with multiple surveys recently published. However, the literature on fundamentally novel principles/materials, is growing at a lower rate, with nanomaterials at the forefront (see for instance the review on nanosensors in healthcare in Gulati et al. (2025)).

Four domains are highlighted in Rong et al. (2025), namely, neuromuscular, motor, post-stroke care, and brain disorder. Within these domains, three classes of technologies were identified, namely, electrical actuators/stimulators (NMES, involving no dof^1^), mechanical/electrical devices (involving mechanical dof), and sensors (EEG, EMG, BMI, MRI, IMU). The class of sensors can be easily divided in internal and external (to the human body). Nowadays, some of these sensors are easily accessible off-the-shelf, at a low cost (see Figure 1).

**FIGURE 1:**
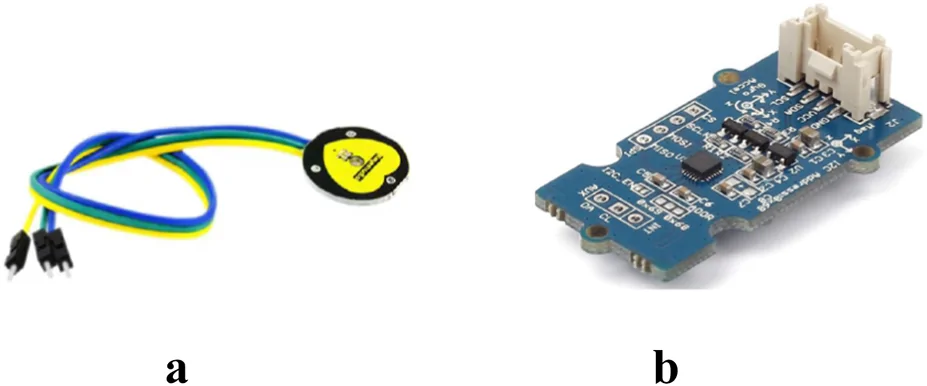
Two examples of low cost off-the-shelf sensors for rehabilitation. **(a)** Keyestudio KS0171-XD-58C EMG sensor. **(b)** Grove 9-dof inertial unit (IMU) sensor.

Rajavenkatanarayanan et al. (2019) reported the use, in multiple studies, of inertial body sensors, electrodermal activity sensors, EMG, ECG, blood volume pulse sensors, vision based motion capture systems, pressure/force plates. Within the robotic devices, exoskeletons and 2D manipulator-like haptic structures are prevalent.

The recent generations of glucose sensors (see for instance Wu et al. (2023)) combine a minimal implantable needle with an external component in direct contact with the skin, in charge of collecting and transmitting the data acquired.

Liu et al. (2025) addressed wearables in human-activity recognition and disease diagnosis. IMU, ECG, EMG, FSR, and temperature sensors are featured, along with AI-based data processing architectures (a large list of LLM-based healthcare architectures is presented).

Porciuncula et al. (2018) points to the decreasing cost trend and the possibility of acquiring large volumes of data, with application in rehabilitation but also is broader health related areas, such as high performance sports. The review emphasizes the roles of IMU, and surface EMG, in stroke, Parkinson and Huntington diseases, osteoarthritis, and running (jogging and sports) populations.

Sengupta et al. (2024) surveyed wearable sensors to monitor motor activities in stroke rehabilitation, namely, accelerometers, gyroscopes, magnetometers, goniometers, pressure sensors, pedometers, EMG, and tilt sensors. The role of machine learning in data processing and prediction of outcomes and assist clinicians is strongly emphasized.

Implantable sensors, are a particular class of wearables, with an estimated market value 15 times bigger than wearables, though with a lower growth rate, Yogev et al. (2023). High cost, regulatory hurdles, patient and physician acceptance, lifetime, and biocompatibility are, among others, relevant factors.

According to Capecci et al. (2024), nine domains are in demand for rehabilitation, namely, robotic devices for upper and lower limbs, non-invasive brain stimulation and brain-computer interface, virtual reality, exergaming, technological systems for balance rehabilitation, function electrical stimulation, telerehabilitation platforms, sensors for monitoring motor functions of upper and lower limbs, and platforms for cognitive rehabilitation.

Internal, or implantable, sensors can monitor physiological or biochemical parameters. Li et al. (2024) reviewed 1D (with a wire structure) implantable sensors and respective most promising materials, namely, hydrogel-based, liquid metal-based, and carbon-based. Suryaprabha et al. (2026) compared materials and performance of wearable and implantable sensors, concluding that, despite the good performances, there are still challenges in long-term reliability, energy autonomy, and data security. Implantable markers, e.g., magnets as in Gherardini et al. (2023) myokinetic interface, can be used to command external devices; the motion of the markers is tracked by a set of externally placed coils.

Circulatory problems can deprive cells of oxygen and nutrients and cause severe health problems. Early detection is thus fundamental for a timely definition of the therapeutics and rehabilitation strategy. There are multiple techniques to analyse the circulatory system (see for instance Matsumoto (2023)). However, the majority is less adapted to body extremities, which are often areas affected. In the case of Diabetic Foot (DF), a common approach is to use an Eco-Doppler probe to test the pedal pulse, directly on the dorsalis pedis. This technique requires technicians skilled in the use of the Eco-Doppler and hence is prone to ambiguous results. Blood circulation is part of the system that regulates body temperature. People with circulatory diseases, such as Peripheral Arterial Disease (PAD), tend to have thinner skin and lower limb temperature, Feller et al. (2022), and hence thermal images may provide useful information to enable the detection of such conditions.

The survey in Wartakusumah et al. (2025) report the validity of applying AI approaches to thermographic images to early detection of DF ulcers and assessment of severity. Multiple approaches were surveyed, namely, SVM, ANN, ResNet50, EfficientNetB0, with reported accuracies between 85% and 100% (for tailored CNN architectures). However, no information on the resolution of the images is reported.

Ilo et al. (2019) used 320 × 240 pixel images of the feet dorsal side in a study with 211 participants. The results pointed to people with neuropathy and neuroischemia having localized regions of higher temperature in high-risk DF participants, meaning difficulties in vascularization.

Thermographic images of the feet sole surface have been used to assess DF conditions in Castillo-Morquecho et al. (2024) in a test with 50 participants. Images were obtained with an off-the-shelf device, allowing resolutions of 160 × 120 or 480 × 360 pixels. The results show a correlation between foot temperature and the average blood sugar level^2^, using a SVM-based strategy for classification.

Overall, the classes of sensors used in rehabilitation appears to be relatively small, with IMUs featured in most papers.

In what concerns actuators, several operation principles are possible, namely, (i) electric, (ii) pneumatic, (iii) soft active materials, and (iv) chemical reactions, among others, Pan et al. (2022). Within each of these classes it is still possible to consider multiple forms, e.g., electric motors can be used directly on the dof or through a system of cables. This is an important project option when volumetry matters, i.e., minimizing the dof volume may require the relocation of the motors and the use of cables to connect the motor and dof.

A cable-driven robot, with a total of seven-dof, is described in Partovi Aria et al. (2023), to help the rehabilitation of patients with spinal chord or stroke injuries. A large gantry-like structure is used to support the weight of the body through cables connecting the gantry with a belt around the user waist. Additional cables connect the waist structure with the lower thighs and shanks.

Besides technology, it is also relevant to consider human factors. A significant gap in the knowledge-to-practice of clinicians is reported in Curtis et al. (2024). Missed expectations of physical therapists relative to wearable technologies, namely, in knee joint rehabilitation, are reported in Williams et al. (2025), along with recommendations of increasing the interactions between the disciplines involved in order to improve acceptance. Perceived usefulness and easy-of-use being relevant factors in the acceptance of technologies.

### 2.1 Kinematics for rehabilitation

Human morphology has a significant number of dof. Human anatomy is composed by 206 bones in adults (with minimal variations, see Neumann and Gest (2019)). From a rehabilitation perspective, the high algebraic complexity of human kinematics is, both, interesting and a challenge. Simplified models, to a few dozens of dof, have been described in the literature. Chung and Hahn (1999) used 36 dof for human walking animation. Cazzola et al. (2017) modelled the body of rugby players with 29 dof. Kudo et al. (2023) used 18 dof just for the lower limbs.

Rehabilitation uses the kinematic redundancies of the human body to enable the creation of devices with simplified kinematics (see for instance Tomita et al. (2018) for a detailed analysis of redundancy, i.e., motor equivalence). Finding strategies and/or designing devices to adjust movements requires a thorough understanding of these redundancies and, often, must be of reduced complexity. Several off-the-shelf devices illustrate such simplified kinematics, e.g., exoskeletons and upper limb rehabilitation devices (see Figure 2).

**FIGURE 2:**
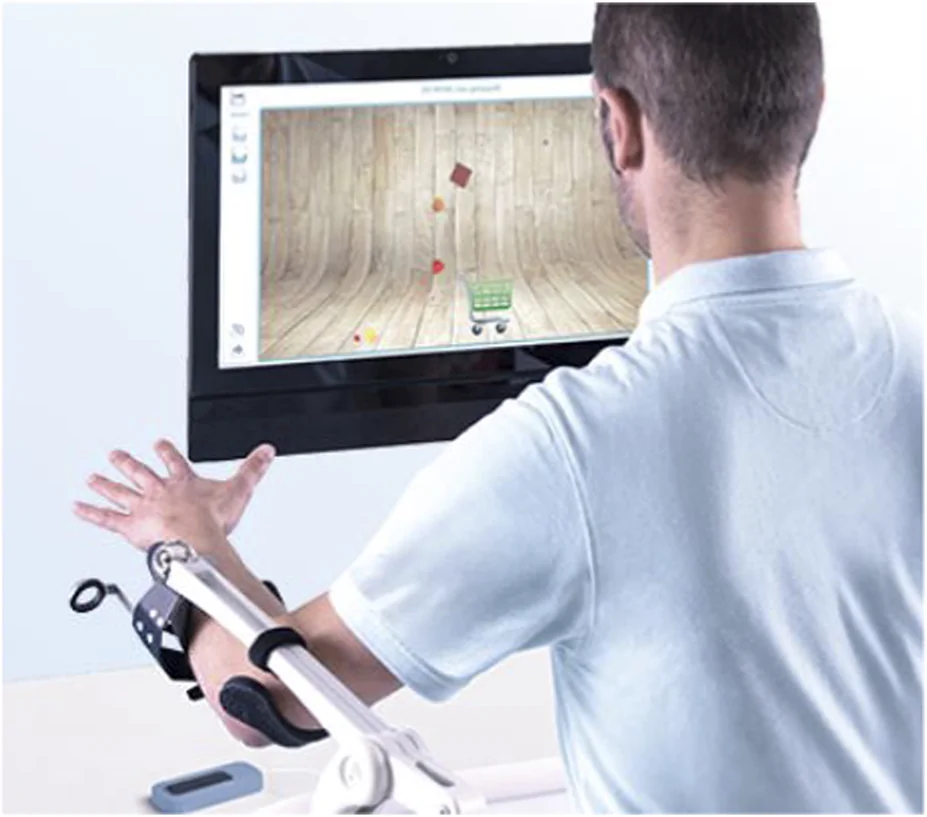
Arm rehabilitation (source: www.btlnet.com/r-lead).

Typical lower limb exoskeletons’ dof range from six-dof, Cruz-Cortes et al. (2024), to eight-dof, Hasan and Dhingra (2022), and ten-dof, Koceska et al. (2013), to fourteen-dof in Woo et al. (2021), among passive and active dof. Upper limbs exoskeletons have been proposed with 2-dof, Durante et al. (2022), 4-dof, Gull et al. (2021), 5-dof, Kang and Wang (2013), and 6-dof, Chen et al. (2014).

In the case of kinematics for knee reeducation, see Brahmia and Kelaiaia (2019), the kinematics is simplified to five-dof (the human knee can be modelled by a six-dof system, Seddiki et al. (2005)).

The waist rehabilitation bed in Ma et al. (2019) has four-dof, with a serial kinematics chain arrangement.

The Robear robot, Jiang et al. (2015), to move people between a bed and a wheelchair, uses two human-type arms, i.e., serial kinematics chains, equipped with force sensors, mounted on a mobile base, with a total of 15 dof.

Overall, kinematics for rehabilitation tends to use low dof to adjust/track the movement of the human body joints in the 3D space. Even though the mechanical complexity of some solutions, low complexity kinematics has natural advantages in terms of the sensing, actuation, and control required.

## 3 Case study: a robotic cane for rehabilitation

Within low dof rehabilitation devices, robotized canes have long been studied, often using unicycle kinematics.

Stramel et al. (2019) presented a three-wheel unicycle kinematics, intrinsically stable, cane to help visually impaired people. The device was equipped with a Lidar to enable detection of the legs and hence adjust the motion based on the position of the user. Additional control was obtained from an haptic (finger touch) plate.

Trujillo-León et al. (2020) described a two-wheel, unicycle, kinematics, equipped with IMU, and weighting 5.7 Kg, resulting in a relatively bulky device. The cane was tested in six participants of 27.5 years old average, with simulated walking impairments.

Liu et al. (2023) proposed a quad-wheel omnidirectional base with a cane pole able to follow a human user by detecting specific tags attached to the calf. The authors report outdoor tests in structured scenarios with healthy users.

Lee et al. (2026) studied the speed control of a unicycle kinematics cane on 24 healthy participants and one stroke patient to improve walking gait symmetry. IMU sensors were used by the participant to provide movement information. The device is claimed to have potential to be as effective as treadmills (which have clinically relevant results).

Yu et al. (2026) described a lightweight cane (1.5 Kg), unicycle based kinematics (intrinsically stable with support wheel), for people with lower limb muscle weakness. The resulting device, equipped with RGB-D camera for capturing user upper-body kinematics and orientation and UWB tags for precise positioning, was tested indoors, successfully, in controlled conditions.

Overall, some of the examples reported in the literature have not been tested with real users, i.e., people with locomotion limitations and/or have a volumetry that might rend its usage difficult in common scenarios, e.g., domestic.

The robotic cane in Neves et al. (2023) (Figure 3) is yet another example of a simple kinematic structure, with a single dof, designed for rehabilitation purposes. From a kinematics perspective, this is an inverted pendulum mounted on top of a unicycle.

**FIGURE 3:**
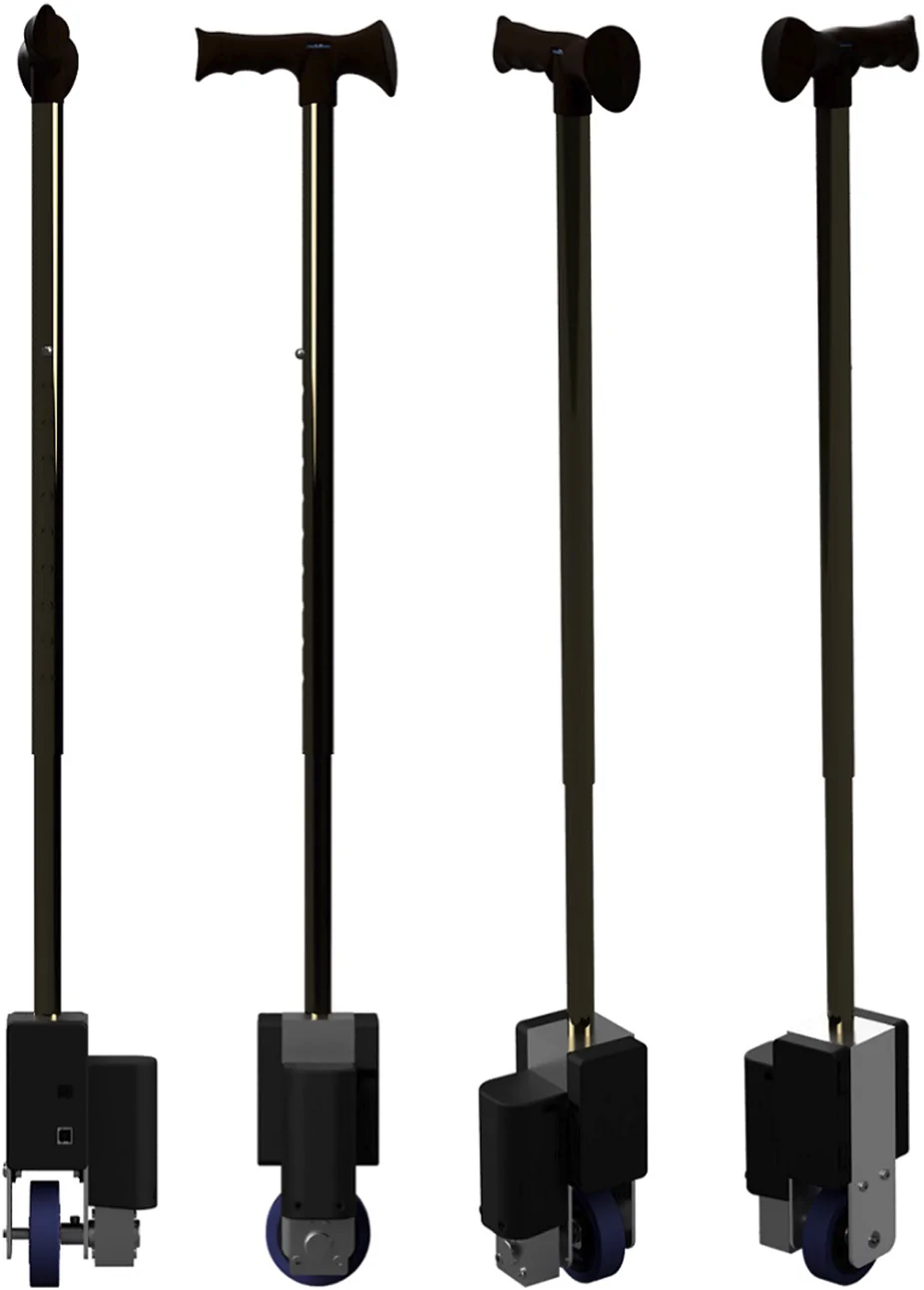
Robot cane (from Neves et al., 2023)

This device matches multiple classes in different taxonomies, e.g., “walking support systems for the elderly” in Noriyuki, (2000), “walking assistance systems”, in Van der Loos and Reinkensmeyer (2008), or “mobility assistance devices” in Speich and Rosen (2004). The expression “smart cane” is commonly used for canes tailored for visually impaired people, as they are able to perceive obstacles in the neighbourhood, though it can also be associated to robot canes.

This project does not use environment perception features to adjust its behaviour. Instead, it is a robotized walking aid for people with limited mobility who may benefit from additional support when walking. The cane is able to adjust its behaviour from user inputs at the handle. It provides an alternative to smart walkers, which, while sturdy, are often heavy and cumbersome. Moreover, besides the use in clinic context, this type of cane can also be used in domestic scenarios, which are often cluttered preventing the use of bulky devices.

Normal canes, as used in physical therapy, are primarily used to preserve the walking gait balance. Moreover, the typical usage assumes a time sequence of fixed ground contact points, without slippage, and the user must move the cane along this sequence. Even though these canes are adjusted to the height of users, trying to preserve physical comfort and minimize the effort, the need to raise and lower the cane, as the walk progresses, requires (i) physical energy and (ii) adaptations to the walking gait in order to preserve balance.

When walking with the help of a traditional cane, humans control the linear velocity of the (tip of the) cane and the respective inclination. Since the tip of cane is only at times in contact with the ground, the overall system, i.e., human and cane, is a variable structure system that switches between (i) a human body with a spherical pendulum (the cane), and (ii) a human body with an additional fixed length link attached to the ground and the hand of the user *via* spherical joints. Learning to use/control the cane is part of the rehabilitation program and, typically, flows from training.

When moving to a robot cane there is a single system, of fixed structure, to control, i.e., the cane never leaves the ground. The orientation of the cane and the linear velocity of the ground tip must be controlled to adjust to the user walking gait and provide support. However, there is only one control variable, i.e., the rotation of the wheel, and hence a tradeoff between the orientation error and the linear displacement must be used.

An evolution of the cane in Figure 3, with a deformable wheel, is described in Ferreira et al. (2026). Figure 4 show snapshots of rehabilitation sessions using this cane^3^. The deformable wheel allows a smoother movement on slightly irregular surfaces without compromising stability.

**FIGURE 4:**
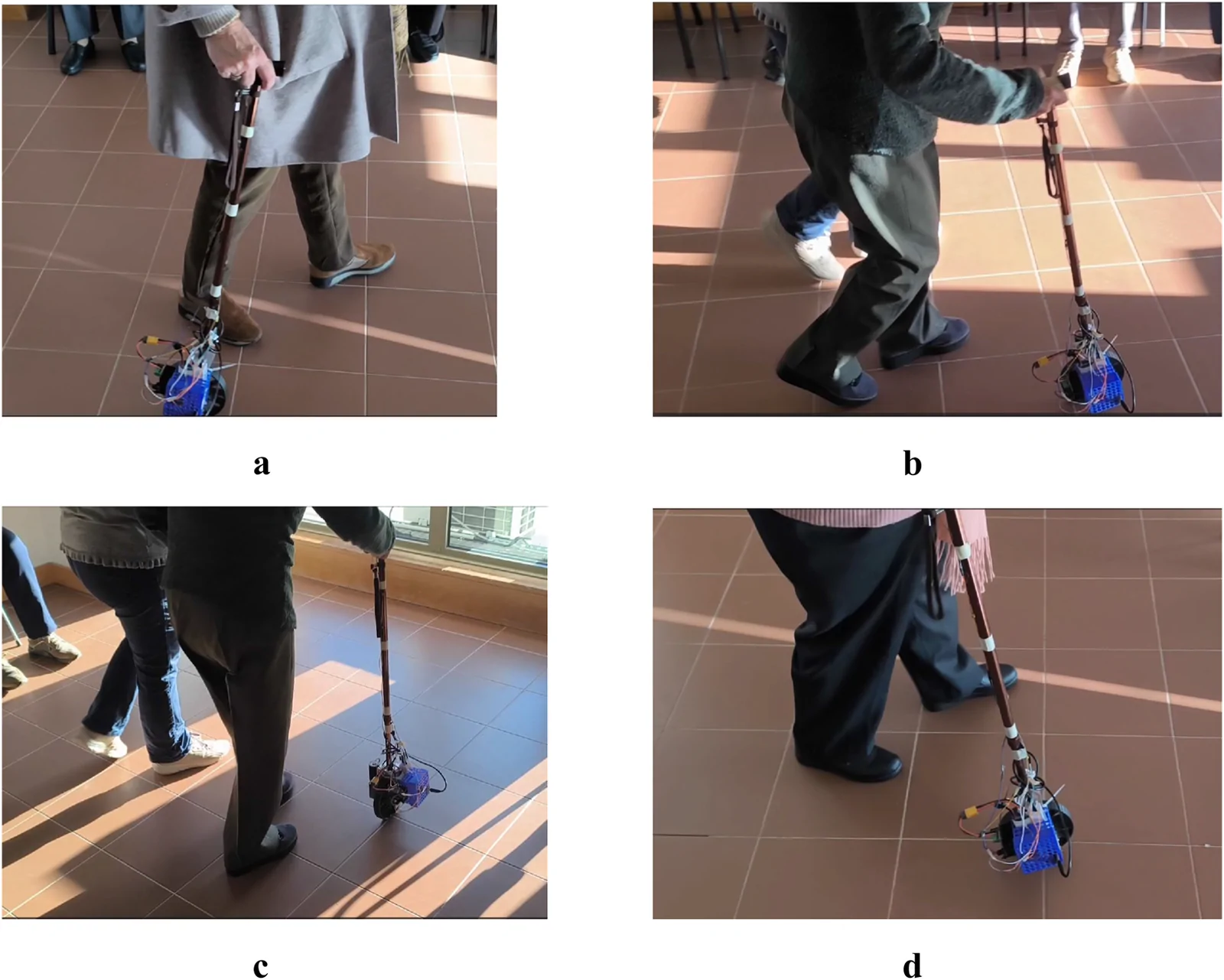
Four snapshots of people in rehabilitation using the robotic cane (images acquired during the experiments reported in Ferreira et al. (2026)). **(a)** User 1. **(b)** User 2. **(c)** User 3. **(d)** User 3.

The design of a robot cane must result from multiple objectives, namely, (i) minimum weight, (ii) small volumetry, (iii) capable of short duration energetic movements, (iv) reduced power consumption, and (v) safe by design (in all circumstances). Subject to these requirements, one might also require that adequate instrumentation is installed onboard.

Clearly, this is a multi-objective problem, and design tradeoffs must be assumed. For example, the weight requirement largely constrains volumetry, and the reduced power consumption constrains the ability to move energetically. Also, volumetry constrains the physical design, namely, imposing the use of small actuators, battery, and allowing limited space for other hardware.

The assessment of the participants was made through a simple questionnaire (to minimize survey fatigue, see for instance Ghafourifard (2024) on this subject), with a three-point Likert question and an open question. The scores obtained in the Likert question were 50%/25%/25%, respectively indicating positive/neutral/negative (or liked/do-not-know/not-liked) attitude towards the robot cane.

## 4 Case study: diabetic foot monitoring

Diabetic Foot (DF) is a condition resulting from Diabetes Mellitus, characterized by poor blood circulation in the feet with the frequent development of ulcerations that may lead to amputations. Given the worldwide prevalence of Diabetes, with an estimated 3.4 million of deaths in 2024, Magliano and Boyko (2025), and a prevalence of DF ulcerations of 18.6 million cases each year, Fuentes-Peñaranda et al. (2025), prevention and risk assessment are paramount.

The generally accepted monitoring of DF is made through a collection of standardized tests, namely, (i) visual inspection (ii) touch, temperature and vibration sensitivity, (iii) pedal pulse detection, and (iv) contact pressure between feet and ground.

Visual analysis rules are explained in Boulton et al. (2008), e.g., dermatological and musculoskeletal assessment, and defined a risk scale covering four categories. Swain et al. (2023) also emphasize the role of visual analysis, pointing to skin colour and hardness, deformities and nail condition.

The survey in Alhasson and Alharbi (2025) reports a growing trend in the use of machine/deep learning approaches effective in the DF image processing, namely, in risk prediction and ulcer classification. Godavarty et al. (2023) recognize the importance of low cost, portable, devices able to perform image analysis to complement human visual assessment.

Visual inspection searches for existing ulcerations and skin condition that may lead to ulcerations are dependent of the experience of the technicians. Figure 5 shows images illustrating the risk assessment annotated by human experts. Texture and discoloration gradients are often primary factors in the risk assessment.

**FIGURE 5:**
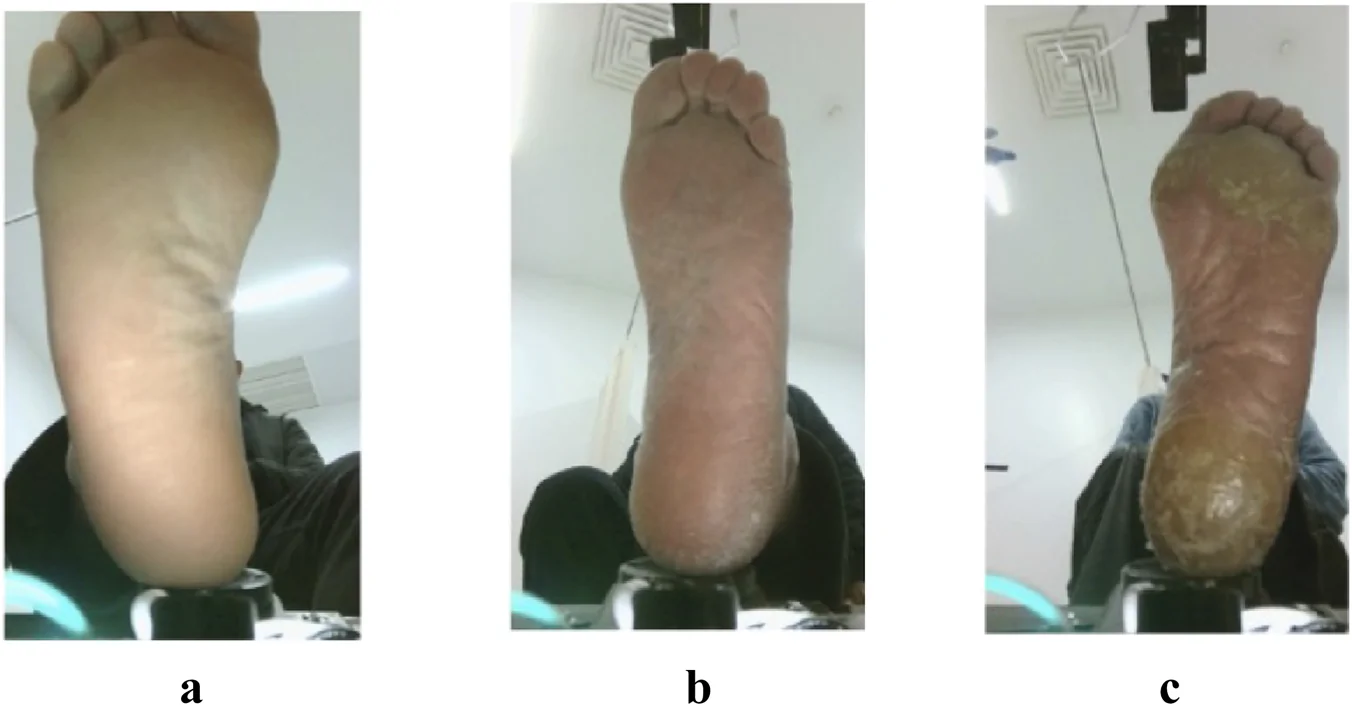
Illustrative images of DF ulceration risk. **(a)** Low risk. **(b)** Medium risk. **(c)** High risk.

The advances in machine learning techniques enable an automatic assessment of these images. Figure 6 show four images obtained for a single patient, within a project on automated DF monitoring (see https://sites.google.com/view/dfaa-pex/home). Figure 7 illustrate an alternative foot image acquisition device (on the left) and the device for foot pressure analysis (on the right). Each image is analised through a ResNet50 CNN, trained with images labelled with the help of Generative AI to yield a risk measure, Nobre and Sequeira (2026). The combination of the risk measures of the four images results in a global metric, yielding a low/medium/high risk label that can be combined with the results from the other DF tests.

**FIGURE 6:**
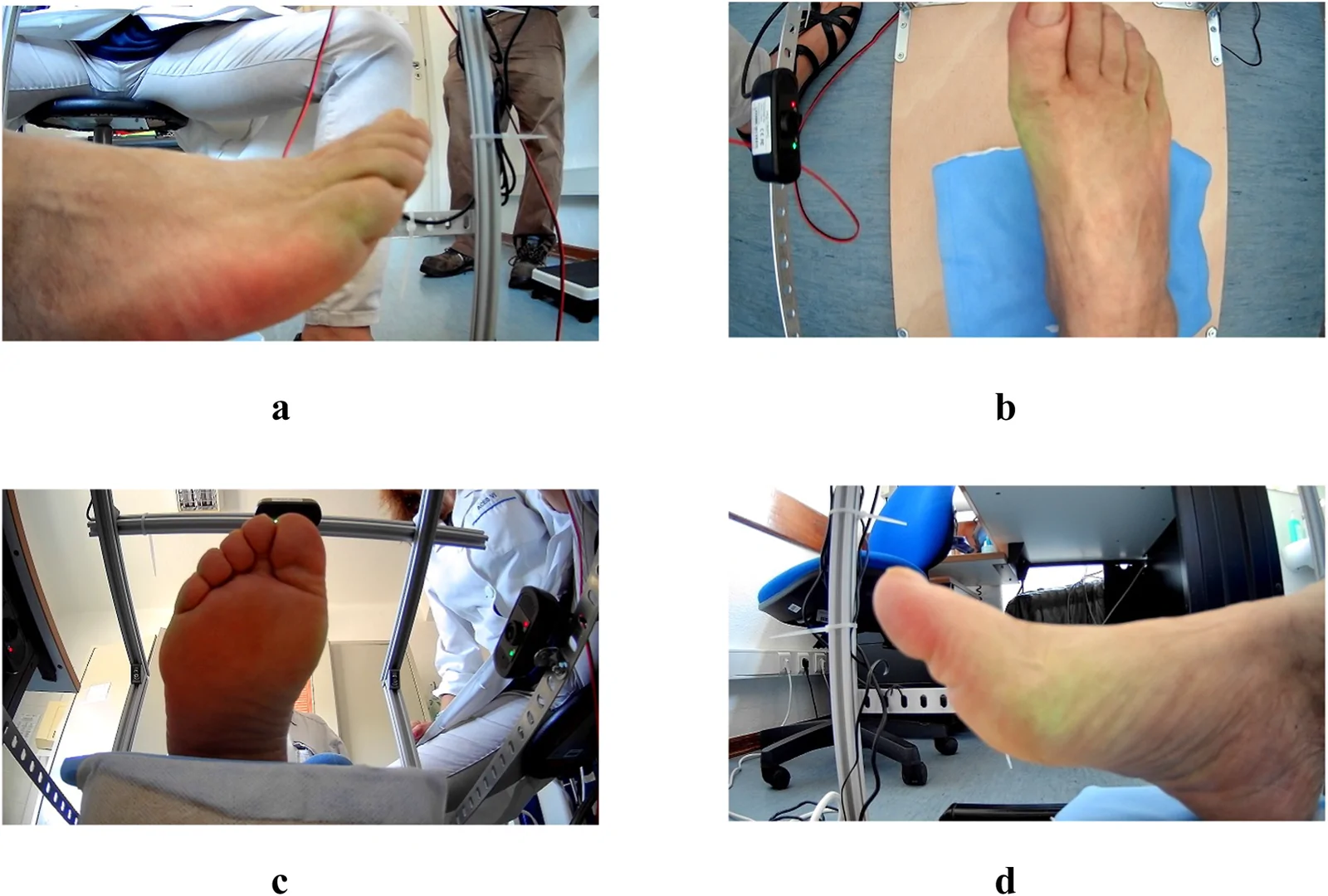
Foot images acquired within DFAA project (https://sites.google.com/view/dfaa-pex/home). **(a)** Right side image. **(b)** Upper side image. **(c)** Bottom side image. **(d)** Left side image.

**FIGURE 7:**
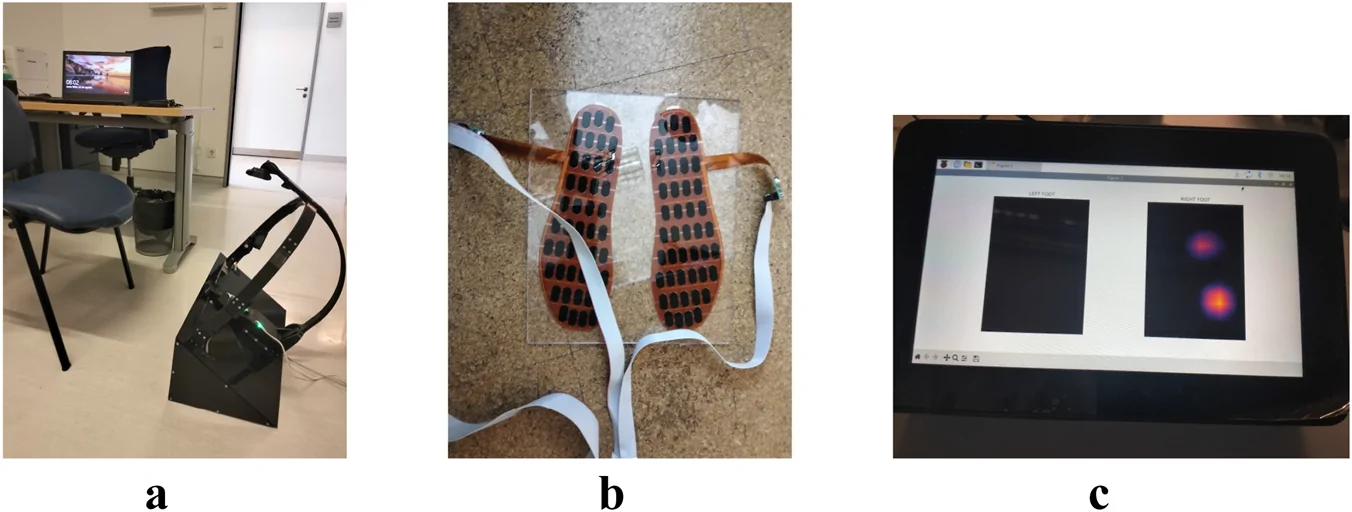
Devices used for image and foot pressure acquisition (two of the standard DF tests). **(a)** Image acquisition device. **(b)** Foot pressure device. **(c)** Foot pressure output.

The dataset, sized 3255 images and three classes, was divided in 70% for training, 15% validation, and 15% testing, with a distribution between classes of 1807, 1294, and 154, respectively for low, medium and high risk. The training occurred for eight epochs, with learning rate $1.5\times 10^{-4}$ , and fine-tuned for an additional 15 epochs, with learning rate $5\times 10^{-5}$ . The images used in this study were obtained using a system of cameras built for the DFAA project^4^ at a ULS hospital.

The natural baseline comparison is with human expert labelling. The Generative AI labelling used achieved an 80.5% over 87 participants.

The original images are resized to 224 × 224 pixels, and were obtained indoors (in a Healthcare institution) where the lighting conditions can exhibit some (minor) variability. A pre-processing stage removes the majority of the background before feeding the images to the CNN.

The F1-score achieved for each label is 76.51%, 72.08%, and 89.74%, respectively for the low, medium, and high risk labels. The combination of this system with the other tests provides a valuable assistant to clinicians. Moreover, the use of Grad-CAM provides a basic explainability tool, indicating which regions of the image contributed the most to the prediction (alternative explainability strategies are possible, e.g., deconvolutional networks, or fuzzy logic rules, see for instance Haar et al. (2023), though the activation map generated by the Grad-CAM yields a visually intuitive image/explanation). Figure 8 shows an example of the interface front-end.

**FIGURE 8:**
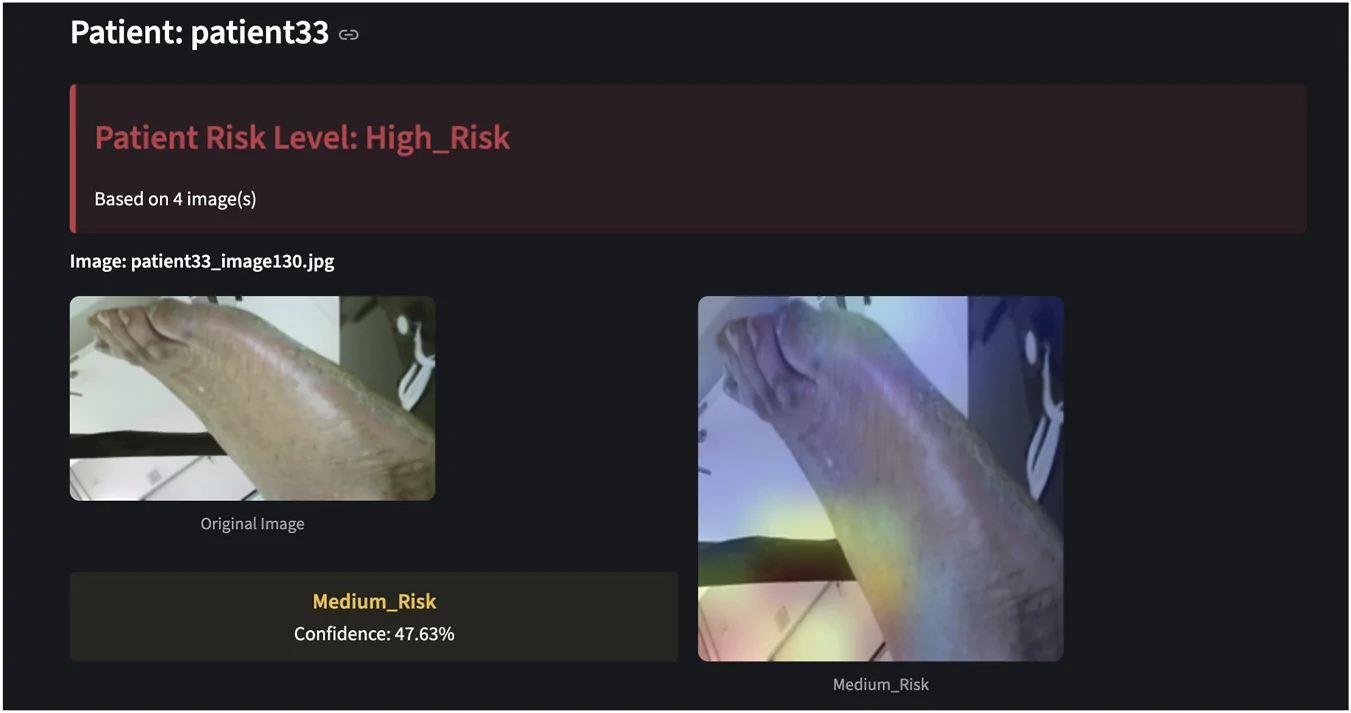
Visual analysis of foot images with DF ulcer risk prediction and Grad-CAM explainability (on the right side foot image).

The Streamlit App developed to generate the report shown in Figure 8 is publicly available from the DFAA project webpage.

## 5 Case study: epilepsy trait detection

The power of AI-based data processing is also shown in an epilepsy trait detection application from sampling EEG images. The visual analysis performed by MD clinicians is strongly influenced by experience, to separate noise from the useful information. Still, the list of features considered by neurologists is large (see Butler et al., 2025) and structured approaches to the reading of EEG, namely, for beginners, has been a topic addressed in the literature, Arbeu-Reys et al. (2023).

Gallotto and Seeck (2023) recognized EEG with a normal appearance may already contain epileptic traits and reviews epilepsy EEG-based biomarkers such as high-frequency oscillations, spikes, brain connectivity, and a microstate representation of the brain. The authors also recognize the usefulness of machine learning to recognize the biomarkers from EEG, reporting accuracies between 86% and 99%.

Alalayah et al. (2023) point to the time consuming of the visually analysis performed by MDs. For the early detection of epileptic seizures, multiple technologies, e.g., K-nearest neighbours, decision trees, random forests, and multi-layer perceptron, were tested and accuracies in the range 98%–99% obtained, on a dataset with 11500 instances.

Using graph convolutional networks Tian and Zhang (2025) detected epileptic seizures with accuracy around 68%.

Using a three layer CNN, fed with small size images (28 × 28 pixels) sampled directly on the EEG, trained and validated on a dataset of 35 abnormal and 85 normal EEGs, Sequeira et al. (2025), report a 77% median accuracy in 909 runs, with precision 0.9 and recall 0.7, which is an interesting result for a recommendation system. Figure 9 show images from the dataset used. The small size of the samples used matches the human trend of inspecting the EEG by sampling the curves for multiple frequencies, e.g., <4 Hz, 4–8 Hz, 8–13 Hz, and >13 Hz, sharp peaks, and asymmetries (see for instance Arbeu-Reys et al. (2023); Amer and Belhaouari (2023) for examples of medical conditions that can be detected by a visual inspection of EEG). Ten sample images per participant (i.e., raw EEG image), randomly selected were used.

**FIGURE 9:**
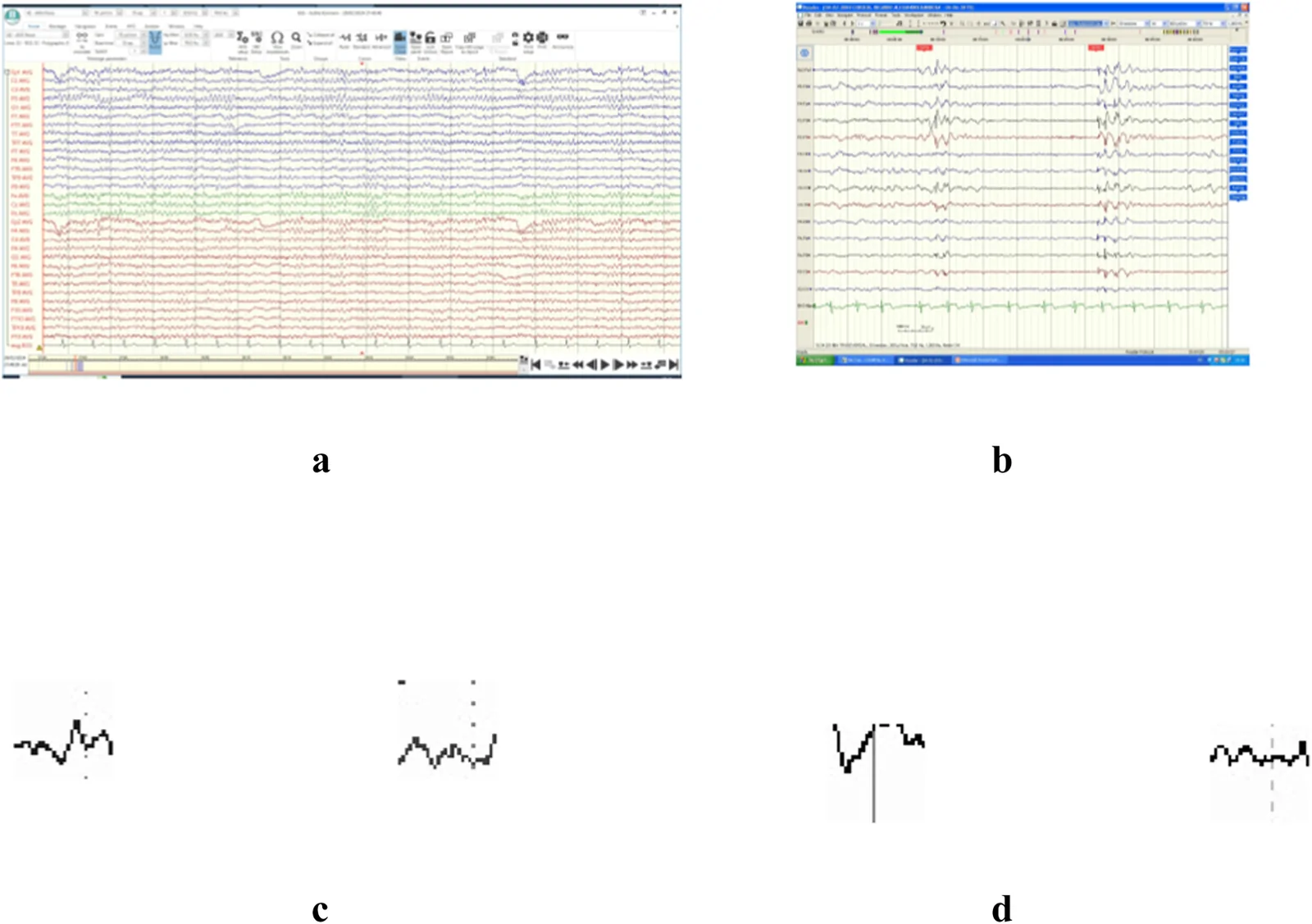
Using machine learning to assist in the detection of epileptic traits. **(a)** Typical raw EEG from a normal patient. **(b)** Typical raw EEG from a patient with epileptic traits. **(c)** Samples from the raw EEG images in the dataset–Participant without epileptic traits. **(d)** Samples from the raw EEG images in the dataset–Participant with epileptic traits.

Figure 10 shows two examples of misclassified samples. Note that the lefthand sample shows pieces of two curves, potentially disturbing the classification. On the righthand side, a seemingly normal sample was misclassified, illustrating, as referred in Gallotto and Seeck (2023), that epileptic traits may be “hidden” in apparently normal EEG.

**FIGURE 10:**
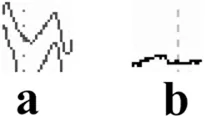
Examples of misclassifications. **(a)** Normal sample classified as epileptic. **(b)** Epileptic sample classified as normal.

The interquartile range obtained, IQR=0.0883 , is fairly low, meaning a high concentration of values around the median, i.e., low dispersion.

## 6 Discussion: human factors and the future

### 6.1 In the real world, outside the lab

Medical experimentation has been at the forefront of protocol design, namely, in what concerns the development or assessment of new medicaments where there are intrinsic constraints to the introduction of biasing human factors, e.g., health safety. In healthcare scenarios standardized protocols are commonly used (see for instance the template in Stoeklé and Hervé (2025), with a flavour in social sciences but adaptable to other domains) and for any new protocol it is often necessary to measure the compliance with existing ones (see, for example, Wilbik et al. (2023) on using fuzzy sets to aggregate multiple measures and determine compliance). Standard practices in protocol design include, among others, the training of study personnel (see Pannucci and Wilkins (2010) or Khan and Khan (2023) for definitions of bias and typical phases in protocols in the clinical context). This is also valid in rehabilitation contexts.

However, the effectiveness in the application of the protocol in a real, non-controlled, environment might be significantly lower than in a controlled environment, e.g., with the clinicians reporting only a subset of the required data, or an image is acquired from an inadequate (less informative) angle, or under poor lighting conditions, or even it is not practical/possible to have people supervising data acquisition. Multiple human factors play a role here and, hence, the poor quality of the data must be considered as a real world characteristic, instead of noise/disturbances, which can, potentially, downgrade the assessments when compared with highly controlled (in-lab) experiments. Therefore, performance assessment in rehabilitation context must be assessed primarily in real world scenarios.

When humans interact with a novel product/device acceptance is often high at the beginning decreasing afterwards. This is called the novelty factor (see Oyman et al. (2022) on multiple factors affecting acceptance) and it easily biases any arguments on acceptance, namely, when experiments have a short timespan, as is the case in in-lab experiments.

In the rehabilitation domain, ethics might also be a barrier to the development of new ideas, namely, being difficult to accept informed consents, equity concerns, or even economic concerns, Shirozhan (2026). For example, participants tend to dislike lengthy informed consents, lengthy evaluation questionnaires, or lengthy explanation sessions, and may introduce answers biases (see Ghafourifard (2024) on survey fatigue). The robot cane use case is an example of a typical experiment where standard protocols may hinder acceptance–elderly people tend to be fragile and less patient to technology development errors and long surveys. Also, patient acceptance reported in Cao et al. (2025) is higher than that in Klaic et al. (2022), which prompts the conclusion that socio-cultural may represent a significant role given that each of the studies took place in different socio-cultural environments. Therefore, even though the standardization of protocols is important for reproducibility and comparison of experiments, namely, in the lab, it is important to note that real world acceptance is often constrained by factors unaccounted for in-lab, such as those referred above.

In addition, understanding the acceptance of robotics technologies from patient and therapist perspectives is critical. For example, Klaic et al. (2022) report a low acceptance by clinicians, despite the good research indicators, with the perception of safety, training, and maintenance concerns being influential. Moreover, migrating technology from the lab to the real world is also subject to generic bias, e.g., low prices (as induced by low cost production) may induce/reinforce a perception of low quality, Seitz et al. (2026), or negative properties being more valued than positive ones, Frank et al. (2023), thus requiring a careful introduction.

### 6.2 Future and challenges

The paper aims at illustrating how low cost technologies can contribute to rehabilitation, primarily, and to associated domains, namely, health monitoring. The global economics diversity is a major drive to a technology adoption drive. Instead of considering the examples presented in the paper as benchmarks, the reader is urged to match real world situations, such as the ones illustrated, and promising technologies. Lab experiments, though certainly useful for initial assessments, are often not realistic enough and not a replacement for the real world. Robotics literature has long identified this problem, see for instance Kanda and Ishiguro (2013). Similarly, in the design/acquisition of image datasets, Goldman and Tsotsos (2024) point to the fact that images without enough information, e.g., generated artificially, may compromise performance statistics “in the real world”, a scenario with similarities with the “model collapse” of AI models.

Confounding factors, e.g., lighting, or ambient temperature, had reduced or no influence in the results presented. Devices to acquire RGB and thermographic images are expected to be installed in structured or semi-structured areas, such as hospitals and pharmacies, where such biasing factors may be controlled.

The use cases presented fall in the intersection between rehabilitation and health monitoring and allow straightforward conclusions, namely, (i) despite the kinematic complexity of the human body, very simple kinematics can yield useful systems, (ii) AI-based techniques, definitely, have a role in rehabilitation, helping technicians to analyse data, and accounting for the variability of the human body and health conditions.

The challenges arise, mainly, in the agile development of experiment protocols which should distinguish between medical viability studies and certification experiments (see, for instance, Desai et al. (2024) on the possible approaches to address the sluggish diffusion of innovation in healthcare).

## Footnotes

1 Acronym for degrees-of-freedom.

2 Glycated hemoglobin test.

3 Experiments performed at two geographically separated branches of a ULS - acronym for Unidade Local de Saúde, or Local Health Unit - of the Portuguese Healthcare System; the participants were duly informed on the purpose and sign informed consent; all data acquired was anonymised at acquisition time.

4 Diabetic Foot Automated Assessment, project 2023.12465.PEX, from Fundação para a Ciência e Tecnologia, Portugal, https://sites.google.com/view/dfaa-pex/home.
